# A combined experimental and computational study of the effect of electron irradiation on the transport properties of aromatic and aliphatic molecular self-assemblies

**DOI:** 10.1039/d2na00040g

**Published:** 2022-07-18

**Authors:** Y. Tong, M. Alsalama, G. R. Berdiyorov, H. Hamoudi

**Affiliations:** Qatar Environment and Energy Research Institute, Hamad Bin Khalifa University Doha Qatar hhamoudi@hbku.edu.qa

## Abstract

Intermolecular cross-linking through electron irradiation is proven to be an effective tool to improve the mechanical and electronic properties of molecular self-assembled monolayers (SAMs), which is known to be a key player for material nanoarchitectonics. Here we study the effect of electron irradiation on the electronic transport properties of aromatic 5,5′-bis(mercaptomethyl)-2,2′-bipyridine (BPD; HS-CH2-(C5H3N)2-CH2-SH) and electron saturated 1-dodecanethiol (C12; CH3-(CH2)11-SH) molecules self-assembled on an Au (111) surface. We could not create any successful junctions for transport measurements for the electron irradiated C12 SAMs due the deterioration of such molecules with electron saturated nature. For the aromatic molecules, the electron bombardment results in significant reduction of the current despite the electron irradiation-induced intermolecular cross-linking, which should create extra transport channels for charge carriers. The current rectification also reduces after the electron bombardment. In order to interpret the experimental results and give right diagnostics behind the decrease of the current through the junction after electron irradiation, we supplement the experiment with quantum transport calculations using Green's functional formalism in combination with density functional theory. The simulation results show that the reduced current after electron irradiation can be related to the detachment of the molecules from the gold substrate and reattachment to other molecules. The formation of diamond-like structures due to intermolecular-cross linking can also be the reason for the reduced current obtained in the experiments. We have also considered the case when the BPD molecules get broken-conjugated due to the attachment of extra hydrogen atoms to the carbon backbone of the molecule. This structural modification also results in a significant decrease of the current. These findings can be useful in understanding the processes during the electron irradiation of molecular SAMs.

## Abstract 2

Intermolecular cross-linking through electron irradiation is proven to be an effective tool to improve the mechanical and electronic properties of molecular self-assembled monolayers, which is known to be a key player for material nanoarchitectonics.

## Graphical abstract

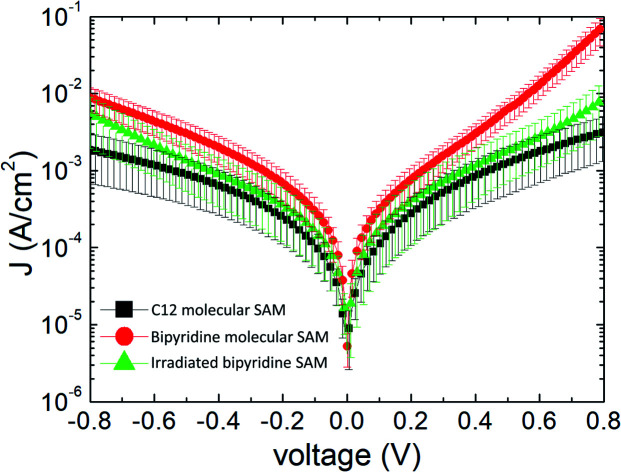

## 1. Introduction

Molecular self-assembly has great potential in creating novel nanoarchitectonic materials with exceptional functionalities to advance in molecular nanotechnology.^1–3^ In particular, self-assembled monolayers (SAMs) of conjugate organic molecules exhibit interesting electronic properties such as selective electronic transport through different molecular orbitals.^4^ The stability of SAMs^5^ and, consequently, the operational stability of SAM-based devices^6^ can be significantly increased by intermolecular cross-linking using, *e.g.*, electron radiation. The structural changes occurring during electron irradiation^7,8^ have a direct impact on other properties of SAMs, such as the electronic structure and charge carrier transport across the molecular junctions. This effect is more pronounced in the case of SAMs of aromatic molecules, for which electron irradiation leads to extensive cross-linking.^9–11^ For example, spectroscopy measurements supported by first-principles calculations show that electron irradiation leads to the reduction of the HOMO–LUMO gap of molecular SAMs.^12,13^ Experiments also show that^14–16^ the resistance of SAMs from aromatic thiolate molecules increases by several orders of magnitude depending on the dose of electron radiation. The latter was related to the increase of the contact resistance due to the combined effect of partially deteriorated anchoring of the molecules to the substrate and irradiation-induced adsorption of airborne molecules at the interface.^16^ The experimental results in ref. 16 about the effect of electron irradiation on the transport properties of aromatic molecular SAMs are described using Simmons' equation with two parameters: the attenuation factor and contact resistance.^17^

In this work, we study the effect of electron irradiation on the transport properties of aromatic 5,5′-bis(mercaptomethyl)-2,2′-bipyridine (BPD; HS-CH2-(C5H3N)2-CH2-SH) and electron saturated 1-dodecanethiol (C12; CH3-(CH2)11-SH) molecules self-assembled on a gold (111) surface. We found that electron irradiation results in the reduction of the current through SAMs of BPD molecules. C12 molecules were highly deteriorated after electron irradiation due to the electron localization nature. Our first-principles quantum transport calculations show that the reduced current can be related to the detachment of the molecules from the gold substrate and consequent reattachment to the neighboring molecules. The formation of diamond-like structures and broken-conjugated structures due to the electron irradiation can also result in current reduction through the junction. Attachment of extra hydrogen atoms to the carbon backbone of the BPD molecule (*i.e.*, broken-conjugation) during the electron irradiation can also result in the reduced current through the molecule.

## 2. Methods

### 2.1. Sample preparation

All the chemicals and solvents were purchased from Sigma Aldrich and used for sample preparation without further purification. Pure ethanol and hexane were degassed under a nitrogen gas flow for 30 min prior to their use. Gold (111) on mica substrates was purchase from PHASIS Switzerland, rinsed with absolute ethanol and dried under a nitrogen gas flow prior to use. SAM structures are created following the procedure described in ref. 2 and 18 as follows. C12@Au SAM was prepared using 1 mM solution of C12 in absolute ethanol. The solution was degassed for 30 minutes before inserting the gold substrate. The system was kept for 4 hours, under reduced light conditions and a nitrogen blanket. After that, the gold substrate was washed three times with absolute ethanol and dried with nitrogen gas. BPD@Au SAM was prepared by using 1 mM solution of BPD in hot hexane (60 °C). The solution was degassed for 30 minutes and maintained at 60 °C before inserting the gold substrate. The system was kept for 15 min, under reduced light conditions and a nitrogen blanket. After that, the gold substrate was washed one time with hexane and three times with absolute ethanol, and then dried with nitrogen gas. Surface analysis and electrical measurement of the studied SAMs were performed immediately after the sample preparation, to avoid the oxidation of the adsorbed molecules.

### 2.2. Photoemission

The photoemission measurements were performed on a standard Thermo Fisher ESCALAB 250XI type XSP platform. A monochromatic Al Kα anode X-ray beam of 1486.6 eV was used with an energy resolution of 0.5 eV. The XPS spectra were obtained with a normal emission and a beam incident of 45° to the surface normal. All the energy values were calibrated with respect to the Au4f located at 84 eV. The irradiation exposure to the sample is performed in the Reflective electron energy loss spectroscopy (REELS) mode with an electron beam of 1000 eV. The exposure time was 30 min.

### 2.3. Transport measurements

The molecular junctions are created by depositing EGaIn on top of SAM structures. We formed junctions containing each type of SAM on as deposited Au substrates or Au on mica for the crosslink sample. We formed 10 junctions on each sample with a 0.2 s delay and in steps of 75 mV from −0.8 V to +0.8 V (higher voltage will oxidize the EGaIn top-electrode and form Schottky bias). We collected ∼200 traces for each SAM. Using the obtained *I*–*V* characteristics, we have calculated the diode figures of merit (resistance, nonlinearity, responsivity, and asymmetry) as:
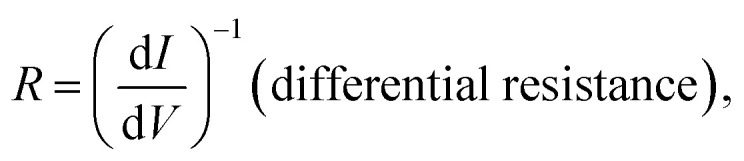

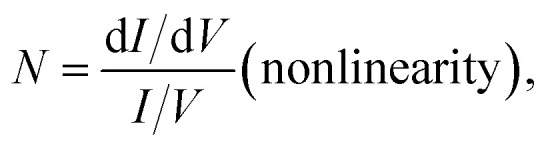

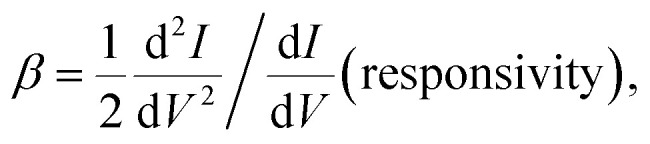

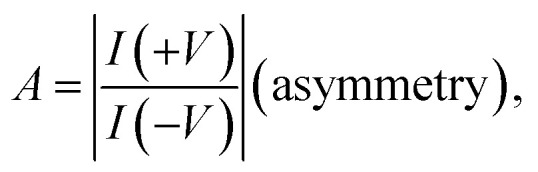
where the asymmetry is defined as the forward-to-reverse current ratio.

### 2.4. Quantum transport calculations

Our model systems consist of either BPD or C12 molecules attached to gold (111) electrodes (see Fig. 4). The electrodes are modeled as a semi-infinite extension of a gold supercell of size 7.11 Å. Geometry optimization and electronic structure calculations are conducted using density functional theory (DFT) within the generalized gradient approximation of Perdew–Burke–Ernzerhof (PBE) for the exchange–correlation energy.^19^ 5 × 5 × 150 Monkhorst–Pack *k*-point Brillouin zone sampling was used in the simulations.^20^ Non-bonded van der Waals interactions, which are important for metal–molecule junction formation,^21^ are taken into account using Grimme's PBE-D empirical correction.^22^ All atoms are described using double-zeta-polarized basis sets. The convergence criterion for Hellman-Feynman forces was 0.01 eV Å^−1^ in the simulations. The nonequilibrium Green's function formalism is used to calculate the electronic transport.^23^ The current–voltage (*I*–*V*) characteristics are obtained using the Landauer-Buttiker formula
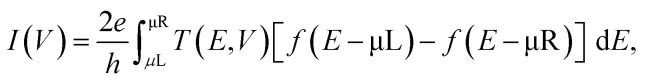
where *T*(*E*, *V*) is the transmission spectrum for a given value of applied voltage (*V*), *f* is the Fermi–Dirac distribution function and μL/μR is the chemical potential of the left/right electrode. The calculations are conducted using the computational package Atomistix toolkit.^24,25^

## 3. Results and discussion

### 3.1. XPS measurements

**Fig. 1:**
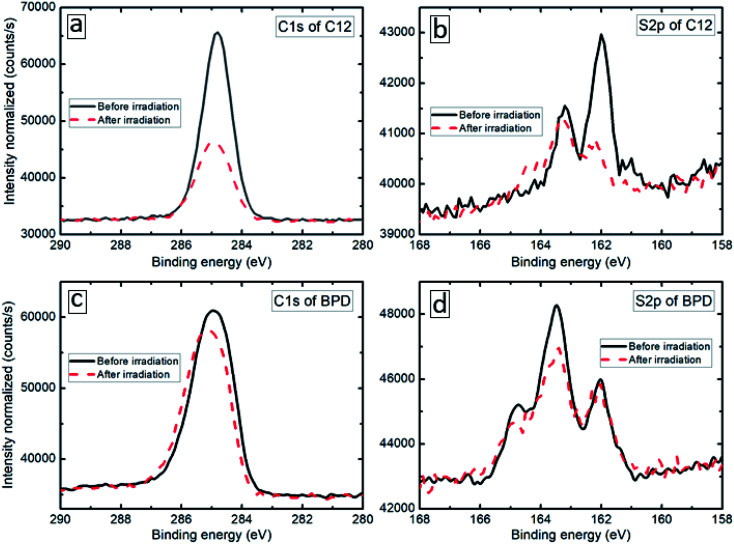
C1s (a, c) and S2p (b, d) core level spectra of C12 (a, b) and BPD (c, d) SAMs before (solid-black curves) and after (dashed-red curves) electron irradiation. The intensities are normalized to the A4f signal.

The XPS spectra of the considered SAM (C12 and BPD) structures are obtained before and after electron irradiation with a particular focus on the deconvolution of the S2p and C1s signals. Fig. 1(b and d) show the S2p core level spectra of C12 (b) and BPD (d) SAMs before (solid-black curves) and after (dashed-red curves) the electron irradiation. The signals of the non-radiated C12 SAM show well defined splitting of the S2p spectrum with the main maximum located at 162 eV (Fig. 1(b)), which corresponds to the thiol sulfur in the S–Au bonding cascade. No clear oxidation component (between 166 and 168 eV) is observed, indicating a good structural integrity of C12 molecules. This is further confirmed by the C1s spectrum (see Fig. 1(a)), which indicates only C–C structure and almost no C–O related peaks expected at 286.5 eV and 288.5 eV, respectively (C–C sp2 at 284.0 Ve, sp3 at 284.8 eV, C–O at 286.5 eV and C=O at 288.5 eV). These findings indicate that all the constituents of C12 are attached to the Au electrode in a uniform manner. The S2p core level signal of the non-radiated BPD SAM is significantly different from the signal obtained for the C12 SAM (compare Fig. 1(b) and (d)). As depicted in Fig. 1(d), the amplitude of the peak at 163.2 eV becomes larger than the one at 161.9 eV, which is not the case for the C12 molecules. This is related to the contribution of the free S end group of BPD. Due to the different nature of the molecules, the C1s spectrum of the BPD molecules is also different from the one obtained for C12 molecules (compare Fig. 1(a) and (c)). For example, the component at 284.8 eV arises from the C–C moieties and C=C in the ring, while a strong shoulder at a higher binding energy of 285.6 eV was attributed to the C–N signal.^16,18^

We then test the film resistance to electron irradiation with a total dose density of 70 × 10^−7^ C cm^−2^ s^−1^ evaluated by using a Faraday cup and an exposure time of 30 min. The XPS results of the considered samples are presented by dashed-red curves in Fig. 1. The C1s signal of the C12 SAM exhibits a great loss in intensity (more than 50%, see Fig. 1(a)) and the S2p signal gets distorted both in intensity and the profile (Fig. 1(b)). As shown in Fig. 1(b), the irradiation induces a significant signal loss of the S2p signal, which clearly indicates that the electron irradiation causes S–Au bond breaking at the interface, leaving the thiol-like fragment unbounded to the substrate. In contrast, the effect of the electron irradiation on both C1s and S2p spectra is much less pronounced in the case of the BPD SAM; the reduction in the C1s signal amplitude is less than 10% (see Fig. 1(c)), whereas no clear variations are obtained in the S2p spectrum (see Fig. 1(d)), except a small reduction of the amplitude of the main peak in the S2p spectrum.^26^ This indicates that BPD shows more resistance to the electron irradiation compared to C12 molecules.

The reduction in the XPS signal intensity can be related to the effective thickness of the molecular SAMs (see *e.g.*, ref. 27). Following the method of ref. 27, we have calculated the effective thicknesses of the self-assembled monolayers before and after the electron irradiation. To determine the effective thickness of the different SAMs, we used the C1s/Au4f intensity ratio, assuming a standard exponential attenuation of the photoelectron signal and the attenuation lengths as reported in the above reference. The calculation results are shown in Table 1. As one can see from these results, the effective thickness of the C12 SAM decreases almost by a factor of 2, whereas the effective thickness of the BPD SAM decreases less than 8%.

**Table 1:** Effective thicknesses of C12 and BPD SAMs before and after electron irradiation

Molecular SAM	Before irradiation	After irradiation
C12	1.200 nm	0.676 nm
BPD	1.470 nm	1.354 nm

**Fig. 2:**
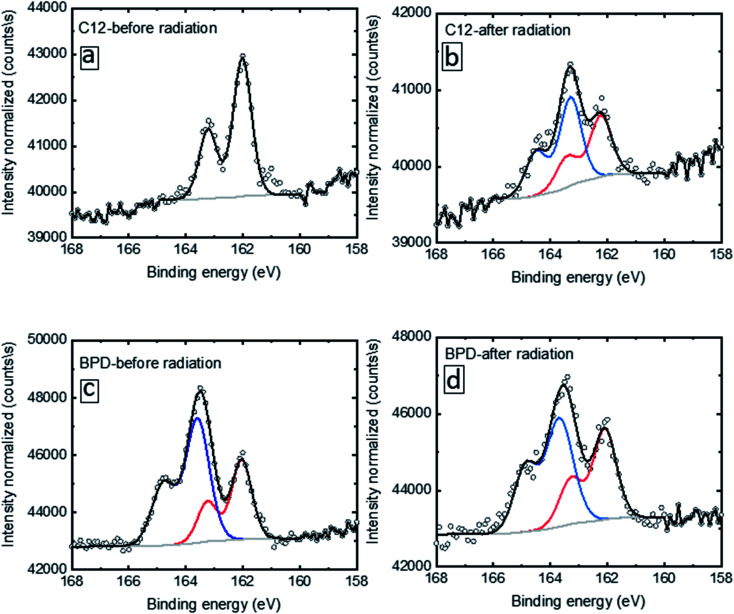
S2p spectra of C12 (a, b) and BPD (c, d) SAMs before (a, c) and after (b, d) electron irradiation. The curves are fitted with a split doublet with the S2p3/2 located at 162.0 eV, corresponding to the S–Au interface signal.

We specifically clarify such evolution by looking into the S2p deconvolution. Fig. 2 shows the deconvolution of the S2p signal for C12 (a, b) and BPD (c, d) for without (a, c) and with (b, d) electron irradiation. In the case of the C12 SAM before radiation (Fig. 2(a)), only one doublet is given with the S2p3/2 located at 162.0 eV, which indicates a well-defined S–Au bond condition. After radiation, extra peaks above 163 eV are observed (Fig. 2(b)), resulting from the decomposition of the molecules upon electron radiation. In the case of the BPD SAM (Fig. 2(c)), we observe the S–Au interface signal with S2p3/2 at 162.5 eV and the top –SH headgroup at 163.5 eV. The former signal was less intense due to the attenuation effect. After radiation (Fig. 2(d)), a slight intensity attenuation is obtained for the 163.5 eV doublet.

**Fig. 3:**
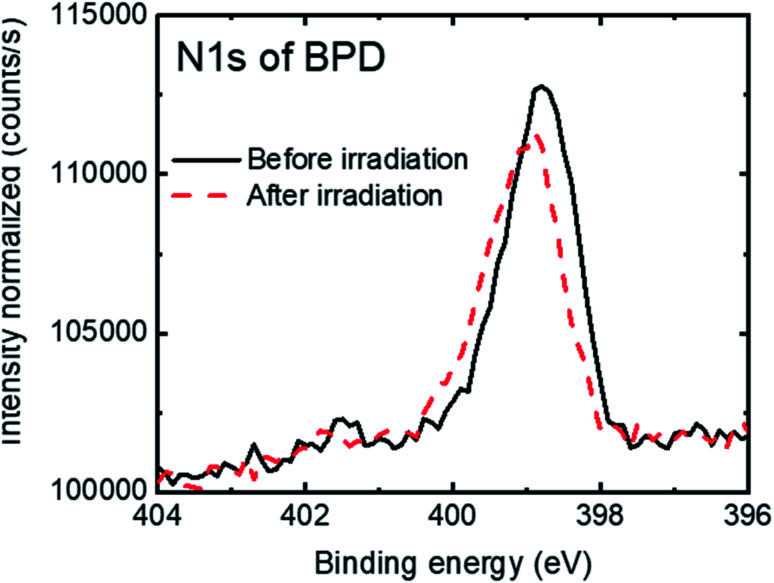
N1s spectra of the BPD SAM before (solid-black curve) and after (dashed-red curve) electron irradiation.

Fig. 3 shows the N1s signal of the BPD SAM before (solid-black curve) and after (dashed-red curve) electron irradiation. We observed a slight attenuation (∼5%) of the N1s signal after electron irradiation. This can be due to the structural reorientation during the electron bombardment and probably also to some molecular detachment from the surface.

**Fig. 4:**
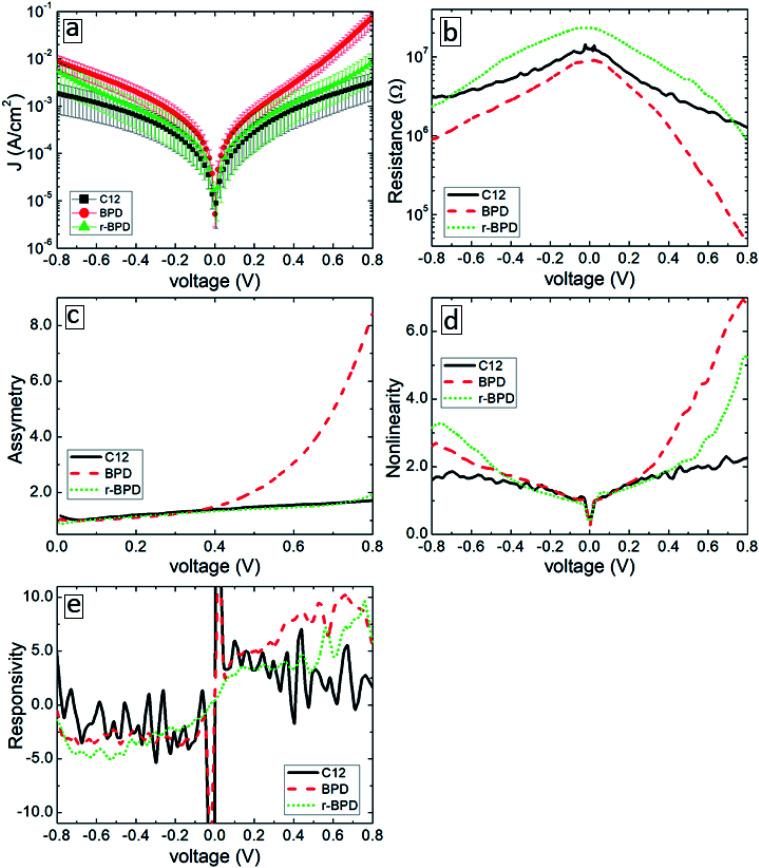
Current–voltage characteristics (a), resistance (b), asymmetry (c), nonlinearity (d) and responsivity (e) of C12 (solid-black curves), BPD (dashed-red curves) and r-BPD (dotted-green curves) SAMs as a function of bias voltage.

### 3.2. Transport measurements

Fig. 4 summarizes the results of our transport measurements, where we present the current–voltage characteristics (a), differential resistance (b), asymmetry (c), nonlinearity (d) and responsivity (e) as a function of bias voltage for C12 (solid-black curves), BPD (dashed-red curves) and irradiated BPD (r-BPD) (dotted-green curves) SAMs. The results are averaged over 10 successful junctions. For the considered range of bias voltages, the largest current density is obtained for the BPD SAMs, whereas C12 SAMs give the smallest current through the molecular junction. The current density in the former system is more than an order of magnitude larger for any given value of the bias voltage. The low value of the current density in C12 molecules can be attributed to the electron saturated nature of C12 molecules with low conductivity. In contrast, BPD molecules have a semimetallic nature with electrons delocalized through the molecular backbone. The electron irradiation results in reduced current in BPD SAMs (see the dotted-green curve in Fig. 4(a)); depending on the value of the applied bias, the drop in the current value can be more than an order of magnitude and the resistance of the BPD-SAMs increases after the electron irradiation (see Fig. 4(b)). A similar effect of electron irradiation was reported in ref. 16. The nonlinearity and responsivity of the junctions also decrease after electron bombardment for most of the bias voltages (see Fig. 4(d and e)). Note that the transport measurements for the C12 sample after irradiation are not presented because we failed to create any successful junctions for the transport measurements.

All considered samples show clear current rectification with forward current being larger than the reverse one. As shown in Fig. 4(c), the largest asymmetry is obtained for non-irradiated BPD SAMs (dashed-red curve). The electron irradiation reduces the current rectification in the BPD system (dotted-green curve); the asymmetry becomes the same as the one obtained for C12 SAMs (solid-black curve).

### 3.3. Transport calculations

**Fig. 5:**
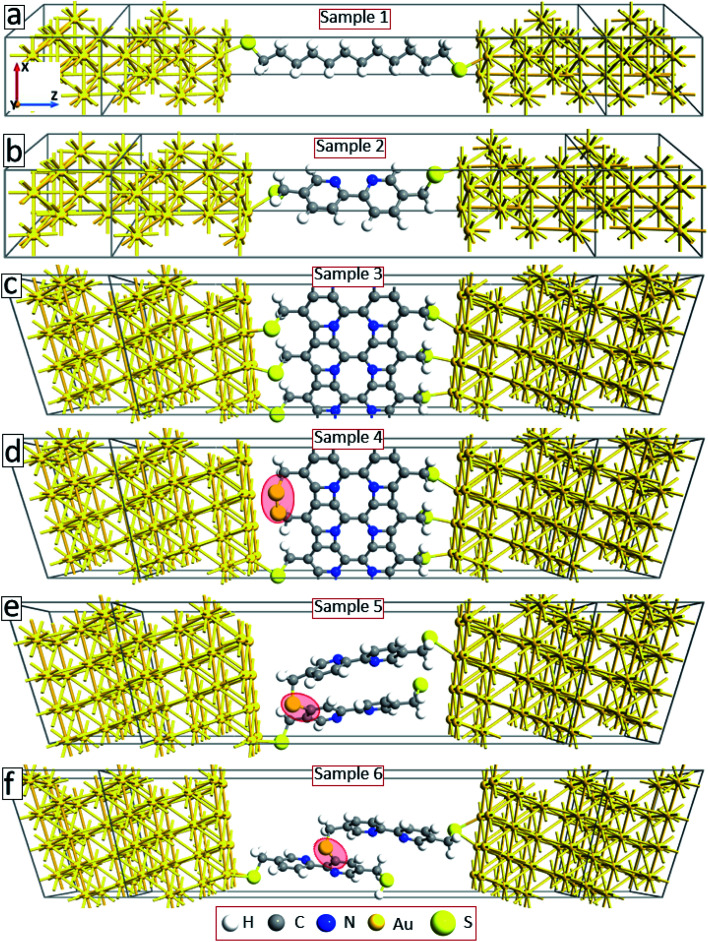
Device geometries: C12 molecule (a), BPD molecule (b), cross-linked BPD molecules (c), cross-linked BPD molecules with defects at the molecule–metal interface (shaded area) (d), and two interconnected BPD molecules through sulfur–carbon covalent bonding (e and f).

**Fig. 6:**
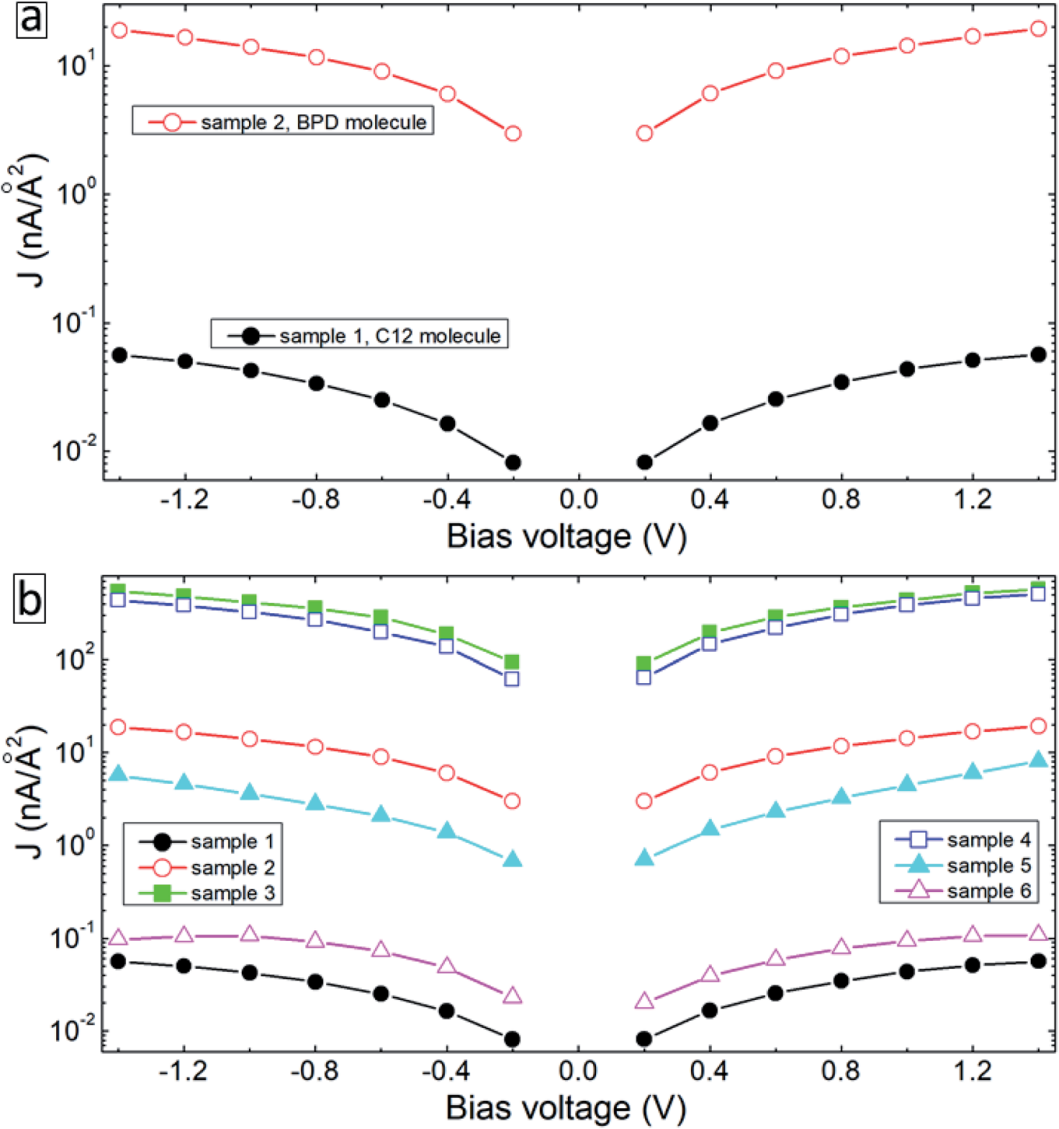
Calculated current–voltage characteristics of the device configurations in Fig. 4.

To explain the experimental findings on the effect of electron irradiation-induced cross-linking on the transport properties of molecular junctions, we have conducted quantum transport calculations using DFT in combination with Green's functional formalism. As a reference, we first construct device geometries consisting of isolated C12 (sample 1) and BPD (sample 2) molecules anchored to gold (111) electrodes through sulfur-gold covalent bonds (see Fig. 5(a and b)). Since we use periodic boundary conditions along the *x*- and *y*-directions, these model structures can be considered as representatives of SAM structures on Au (111) structures. These samples will help us to validate our theoretical approach towards modelling our experimental samples. Solid-black and open-red circles in Fig. 6(a) show the calculated current–voltage characteristics of these two systems. As was obtained in the experiment (see Fig. 4(a)), the BPD sample shows enhanced current as compared to the C12 sample. The difference in the current is more than 2 orders of magnitude in the simulations. Thus, the calculated *I–V* characteristics of these two molecules are in good agreement with the experimental results.

Fig. 5(c) shows our model system for an electron-irradiated BPD SAM with intermolecular cross-linking (sample 3). The calculated *I–V* curve of this system is shown by solid-green squares in Fig. 6(b). It is seen from this figure that the cross-linking of the molecules only on the lateral sides increases the current in the system by more than an order of magnitude, which can be attributed to the creation of extra transmission channels for the electrons through the junction. However, this finding is not in good agreement with the present experiment (see Fig. 4(a)) and available reports in the literature.^16^ The experiments show that the electron irradiation results in reduced current in the system. One of the reasons for the exponential increase of the junction resistance with increasing the dose of the electron irradiation was proposed to be the partial damage of the thiolate anchors.^16^ In order to validate this scenario, we broke the sulfur–gold covalent bonds and created sulfur–sulfur bonds as proposed in ref. 16. Our model system is shown in Fig. 5(d) (sample 4) with the highlighted S–S covalent bond. Such structural changes decrease the current in the system (open-blue squares in Fig. 6(b)). However, the conductance is still larger than the one for the non-radiated BPD system (open-red circles in Fig. 6(b)), which is due to the overlapping between the S orbital and the d-band of the gold substate.

Another proposal for the reduced current in molecular junctions after electron irradiation can be the possible adsorption of the disrobed airborne molecules from the Au substrate after electron irradiation at the radicals–SAM–ambient interface. To check this proposal, we have constructed two model systems where some of the BPD molecules get desorbed from the substrate and reconnect to the neighboring molecules through sulfur–carbon covalent bonds (samples 5 and 6, see Fig. 5(e and f)). Significant reduction of the current is obtained when the sulfur atom of the desorbed BPD molecules is attached to the carbon atom in close proximity to the substrate (sample 5, filled-cyan triangle in Fig. 6(b)). The current reduces by more than 2 orders of magnitude when the sulfur atom forms a covalent bond with the upper phenyl ring (sample 6, open-magenta triangles in Fig. 6(b)). Thus, the desorption and reattachment of the molecules of the SAM can be the origin for the obtained reduction of the current after electron irradiation.

**Fig. 7:**
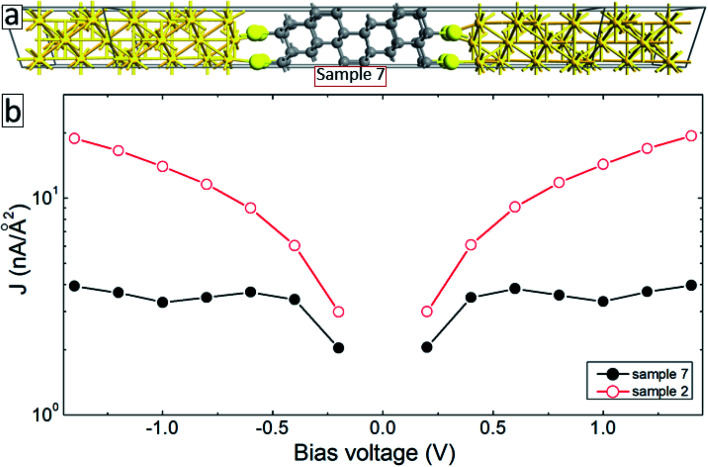
(a) A model system consisting of a diamond structure of carbon atoms sandwiched between gold (111) electrodes (sample 7). (b) *I*–*V* characteristics of sample 7 (solid-black curves) and sample 2 (open-red circles).

The aromatic molecules can also form diamond-like structures during the electron irradiation due to successful intermolecular cross-linking. In order to see how such structural transformation may affect the transport properties of the junctions, we have constructed a model system consisting of a thin layer of the diamond structure of carbon atoms anchored to gold electrodes through thiol groups (sample 7, see Fig. 7(a)). Solid-back circles in Fig. 7(b) show the *I*–*V* characteristics of this system for the same range of applied voltages. The results for our reference system (sample 2) are also shown in this figure (open-red circles). It is seen from this figure that the current density in the system decreases by more than a factor of 4 depending on the applied voltage. Thus, diamond-like structural transformation can also be used to explain the effect of electron irradiation on the transport properties of molecular SAMs.

**Fig. 8:**
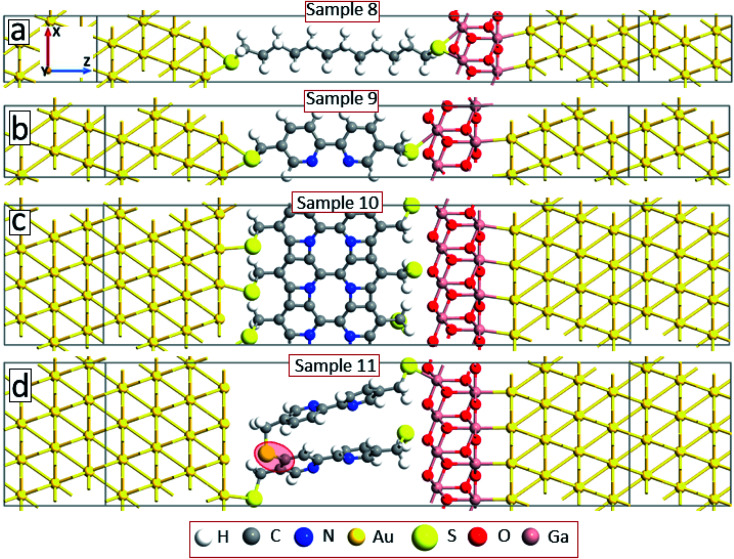
Model systems as in Fig. 6 but with the right electrode modified by inserting a thin layer of Ga_2_O_3_.

**Fig. 9:**
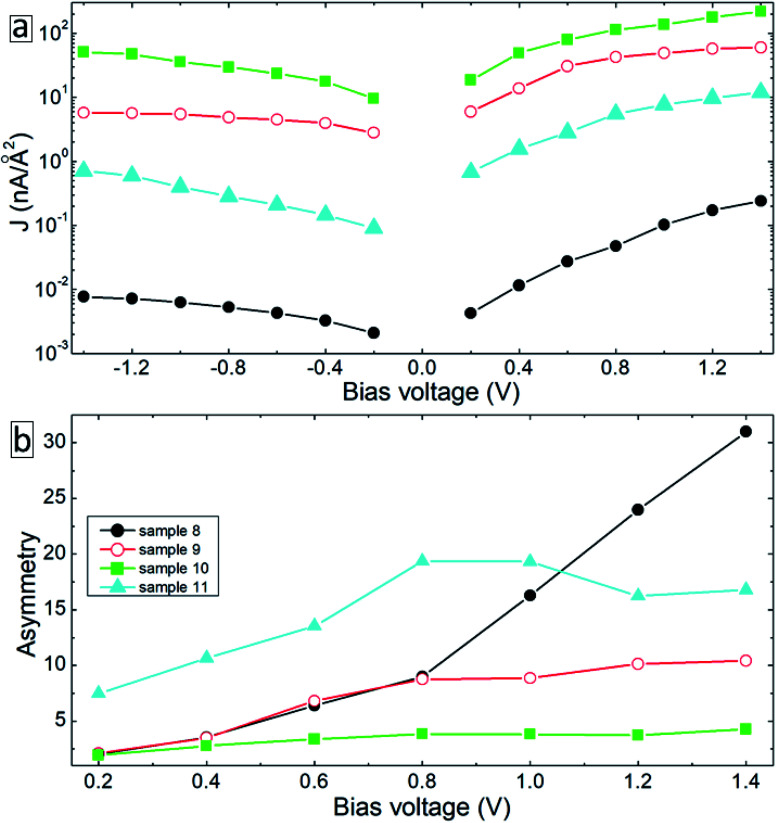
*I*–*V* characteristics (a) and asymmetry (b) of the device configurations in Fig. 8.

Note that due to the symmetry of the molecules and electrode configurations, no asymmetry in the *I–V* curves is obtained for the considered device geometries. Small asymmetry is obtained for samples 5 and 6 (the rectification level is smaller than 1.5) due to the asymmetric arrangement of the molecules with respect to electrodes. The obtained current rectification in the experiments (see Fig. 4(c)) is due to the different electronic properties of the top and bottom electrodes. To obtain better model systems, we have included a thin layer of Ga_2_O_3_ between the molecules and the gold electrode as shown in Fig. 8. We have chosen the Ga_2_O_3_ layer because it is formed when the EGaIn electrode is deposited onto molecular SAMs. Fig. 9(a) shows the *I–V* characteristics of the device structures presented in Fig. 8. For all the considered systems, the insertion of the oxide layer decreases the current in the system for any given value of the applied voltage. However, we obtained the same dependence of the value of the current on the type and arrangement of the molecules: the smallest current is obtained for the saturated C12 molecules (sample 8, solid-black circles), whereas the fully cross-linked system (sample 10) gives the largest current density. Still, the desorption of the molecules from the substrate (sample 11) gives reduced current as compared to the reference system (sample 9) (compare open-red circles and filled cyan triangles in Fig. 9(a)). All the considered samples show clear current rectification with forward current being larger than the reverse one. Fig. 9(b) shows the calculated rectification ratios as a function of bias voltage. Samples 8 and 9 exhibit the same level of rectification for bias voltages up to 0.8 V, starting from which the C12 molecule gives larger rectification. The asymmetry in the *I–V* curves becomes smaller for the system with intermolecular cross-linking (sample 10, filled-green squares). Sample 11 with the asymmetric location of the molecules also gives larger rectification as compared to the reference system.

**Fig. 10:**
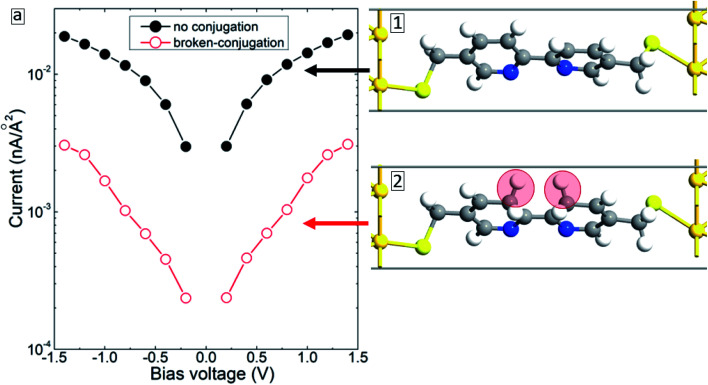
*I*–*V* characteristics of the BPD molecule attached to Au(111) electrodes without (solid-black circles and inset 1) and C-conjugation (open-red circles and inset 2). The additional hydrogen atoms are highlighted in inset 2.

Fracasso *et al.*^28^ studied the transport properties of self-assembly of organic molecules with linear-conjugation, broken-conjugation, and cross-conjugation. Both transport measurements and numerical simulations show that linear-conjugated molecules are significantly more conductive than the molecules with either cross-conjugation or broken-conjugation. Since such structural changes may occur during electron bombardment,^16^ we have considered the case when the BPD molecule gets modified (conjugated) as shown in inset 2 of Fig. 10. The simulation results show that (see open-red circles in Fig. 10(a)) such structural changes result in significant reduction of the current through the molecular junction due to the localization of the electrons in the modified zone. Thus, the formation of broken- or cross-conjugated bonds can also be used to explain the experimentally obtained reduction of the conductivity of aromatic molecular SAMs during the electron irradiation.

## 4. Conclusions

The effect of electron irradiation on the electronic transport properties of aliphatic 1-dodecanethiol (C12) molecular SAMs and 5,5′-bis(mercaptomethyl)-2,2′-bipyridine SAMs is studied experimentally. BPD SAMs exhibit a significant increase in the resistance with a consequent current reduction upon electron bombardment. The electron bombardment causes a significant damage to the C12 SAMs as revealed in our XPS measurements. Therefore, no successful molecular junctions were created for electrical transport measurements. The experimental results are supplemented by first principles quantum transport calculations with the non-equilibrium Green's functional formalism. The calculations show that the formation of diamond-like structures or the detachment of the molecules from the substrate and reattachment to the neighboring molecules can explain the experimentally obtained results about the reduction of the current after electron irradiation. Numerical simulations also show that the current in the system can be reduced significantly when the BPD molecules get broken-conjugated due to the attachment of extra hydrogen atoms to the backbone of the molecule. These findings can be useful in understanding the structural changes in molecular SAMs during electron bombardment for different nanoarchitectonic applications.

## Data availability

Data are available from the authors upon request.

## Conflicts of interest

The authors declare no competing interests.

## Author contributions

H. H., Y. T., and M. A. prepared the samples and conducted XPS and transport measurements. G. R. B. conducted quantum transport calculations. All authors contributed to manuscript preparation. H.H. directed the project.

## Supplementary Material
